# A Simple and Computationally Efficient Approach to Multifactor Dimensionality Reduction Analysis of Gene-Gene Interactions for Quantitative Traits

**DOI:** 10.1371/journal.pone.0066545

**Published:** 2013-06-21

**Authors:** Jiang Gui, Jason H. Moore, Scott M. Williams, Peter Andrews, Hans L. Hillege, Pim van der Harst, Gerjan Navis, Wiek H. Van Gilst, Folkert W. Asselbergs, Diane Gilbert-Diamond

**Affiliations:** 1 Institute for Quantitative Biomedical Sciences, Geisel School of Medicine, Lebanon, New Hampshire, United States of America; 2 Section of Biostatistics and Epidemiology, Departments of Community and Family Medicine, Geisel School of Medicine, Lebanon, New Hampshire, United States of America; 3 Department of Genetics, Geisel School of Medicine, Lebanon, New Hampshire, United States of America; 4 Department of Cardiology, University Medical Center Groningen, Groningen, The Netherlands; 5 Department of Nephrology, University Medical Center Groningen, Groningen, The Netherlands; 6 Department of Cardiology, Division of Heart and Lungs, University Medical Center Utrecht, Utrecht, The Netherlands; University of Texas School of Public Health, United States of America

## Abstract

We present an extension of the two-class multifactor dimensionality reduction (MDR) algorithm that enables detection and characterization of epistatic SNP-SNP interactions in the context of a quantitative trait. The proposed Quantitative MDR (QMDR) method handles continuous data by modifying MDR’s constructive induction algorithm to use a T-test. QMDR replaces the balanced accuracy metric with a T-test statistic as the score to determine the best interaction model. We used a simulation to identify the empirical distribution of QMDR’s testing score. We then applied QMDR to genetic data from the ongoing prospective Prevention of Renal and Vascular End-Stage Disease (PREVEND) study.

## Introduction

The view that the genetic basis of common human diseases can be explained by sequence variation in a few genetic loci has been recently replaced by a new appreciation for the complexity of biological networks and the interplay between proteins that jointly influence phenotypes [1]. The recent advances in high-throughput genotyping techniques have made large quantities of genotype data commonplace in genetic epidemiology studies and therefore have enabled researchers to interrogate the entire genome. Researchers have extensively analyzed single SNP effects for a wide variety of diseases/phenotpyes with variable results, but in most cases with a large proportion of the genetic component (heritability) left unexplained. It has been proposed that these limitations are due to the analytical strategy that limits analyses to only single SNPs [2], and it is therefore becoming more commonplace to assess the challenge of identifying SNP-SNP interactions.

The problem of identifying interactive SNP effects in a case-control study, which can be formulated as predicting binary outcomes, has been studied extensively and has demonstrated great promise in recent years [3]–[5]. Multifactor Dimensionality Reduction MDR was developed as a nonparametric and model-free data mining method for detecting, characterizing, and interpreting epistasis in the absence of significant main effects in genetic and epidemiologic studies of complex traits such as disease susceptibility [3]. The goal of MDR is to change the representation of the data using a constructive induction algorithm to make non-additive interactions easier to detect using a classification method such as naïve Bayes or logistic regression. Comparative studies [6], [7] that use extensive simulations show that MDR has the best performance when the true multi-SNP effects are non-additive [2], [3], [7]–[12].

Despite the fact that MDR has been extended to various settings [13]–[17], there have been few attempts to develop methods that systematically identify SNP-SNP interactions in relation to quantitative outcomes such as body mass index, tumor size and survival time. Because in many cases, analyzing phenotypes as continuous rather than binary outcomes can be more powerful due to large variability in the outcome distribution it is important to develop methods that permit the analyses of continuous traits.

Many methods including Combinatorial Partitioning Method (CPM) [18], Repeated Partitioning Method (RPM) [19] and U-statistics [20] were developed to identify interactions for quantitative traits. CPM and RPM are both computationally intensive and the U-statistics approach is limited to detecting interaction models with main effects. In 2006, Generalized MDR (G-MDR) [17] was proposed to extend the MDR algorithm to be applicable to continuous phenotypes and allow covariate adjustment. In 2011, Model based MDR (MB-MDR) [21] was developed to improve MDR using parametric regression; this method can also be applied to continuous outcomes. However, neither G-MDR nor MB-MDR provides a computationally efficient algorithm that is easy to implement. MB-MDR is implemented in R (http://www.r-project.org/) but it can only search over all one-way and two-way interaction models. G-MDR still requires a dichotomous outcome in the data file. Furthermore, the success rate of each algorithm on continuous outcomes was not evaluated using simulations.

In the current paper, we present an extension of the multifactor dimensionality reduction (MDR) algorithm to detect and characterize epistatic interactions in the context of a quantitative outcome (QMDR). We first present the type-I error and success rate of the proposed method using simulated datasets under different epitasis models. We then present the results of QMDR applied to genetic data from the ongoing prospective Prevention of Renal and Vascular End-Stage Disease (PREVEND) study.

## Materials and Methods

In this section, we introduce T statistics and describe how they can be used in the MDR framework in the context of quantitative outcomes.

### MDR Algorithm

Traditional MDR is a data reduction approach that identifies multi-locus combinations of genotypes that are associated with either high or low risk of disease and uses them to define a new single high/low risk attribute. The general process of defining a new attribute as a function of two or more other attributes is referred to as constructive induction or attribute construction [22]. Constructive induction using MDR for binary outcomes (e.g. case-control status) is accomplished in the following way:

1. Assume there are m SNPs in the dataset; in order to examine a K-order interaction, select K SNPs from the m SNPs.
2. Construct a contingency table using these K SNPs and calculate case-control ratios for each multi-locus genotype.
3. Let R be the ratio of cases to controls in the whole dataset. For each multi-locus genotype, if the ratio of cases to controls exceeds T, it is considered high-risk. Otherwise, it is considered low-risk. Once all genotypes are labeled ‘high-risk’ and ‘low-risk’, a new binary attribute is created constructed by pooling the “high risk” genotype combinations into one group and the “low risk” into another group.

MDR uses a simple probabilistic classifier that is similar to a naïve Bayes classifier [8] to model the relationship between variables constructed using MDR and case-control status. Naïve Bayes classifiers were assessed using balanced accuracy [23]. Balanced accuracy is defined as the arithmetic mean of sensitivity and specificity:
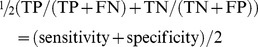
(1)where TP are true positives, TN are true negatives, FP are false positives, and FN are false negatives. For each dataset, MDR evaluates all possible K-way interactions and identifies the best model using balanced accuracy. To determine the K loci that give the best model overall, MDR uses ten-fold cross-validation:

1. Divide the dataset into 10 partitions. Using 9/10 of the data as a training set and the rest as a testing set.
2. Compute the training balanced accuracy for each K-way interaction in the training set.
3. Using the K-way model that has the best training balanced accuracy, predict the case-control status in the testing set.
4. Repeat steps two-three 10 times so that each partition is included in the testing set once.
5. Compute a testing balanced accuracy by using the case-control predictions and actual case-control status for all 10 testing sets. For the K-way models that are chosen from the training sets, record how many times each is identified as the best model (cross-validation consistency).

The best MDR model is selected as that with the maximum testing balanced accuracy and highest cross-validation consistency. The latter is used as a tie-break. If both statistics are tied, then the more parsimonious model is chosen as the overall best model.

### Quantitative MDR Algorithm

Quantitative MDR (QMDR) extends the MDR algorithm described above to work with quantitative or continuous phenotypes. Instead of comparing the case-control ratio of each multi-locus genotype to a fixed threshold R, we propose to compare the mean value of each multi-locus genotype to the overall mean. Constructive induction by QMDR is done as follows:

1. Assume there are m SNPs in the dataset; in order to examine a K-order interaction, select K SNPs from the m SNPs.
2. For each multi-locus genotype combination defined by the K SNPs, calculate the mean value and compare it with the overall mean.
3. If the mean value from the genotype combination is larger than the overall mean, the corresponding genotype is considered high-level. Otherwise, it is considered low-level. Once all of the genotypes are labeled ‘high-level’ and ‘low-level’, a new binary attribute is created by pooling the “high-level” genotype combinations into one group and the “low-level” into another group.

With quantitative outcomes, we cannot use balanced accuracy to characterize the relationship between the QMDR attribute and phenotype. Instead, we compare the outcome between high and low level groups defined by the QMDR attribute using a T-test and then use the T-statistic as a training score to choose the best model. The cross-validation procedure for QMDR is the same as that used in traditional MDR. The difference is that we define the training score and testing score from the T-test (replacing the training and testing balanced accuracy). We use the training score to determine the best K-order interaction model and use the maximum testing score to identify the best overall model. When there is no SNP effect, QMDR attributes from the testing set are equivalent to ones randomly assigned to the high or low level group. Therefore we expect that the null distribution of the testing score follows a normal distribution with mean 0. We can then use an empirical null distribution to estimate the *p-*value of the chosen model. In the next section, we will use simulated data to compare empirical *p-*value and the permutated *p-*value to verify the above hypothesis.

### Simulations

To demonstrate the strength of the proposed method, we designed two simulations: Simulation I focused on estimating the threshold for 5% type I error using a quantitative outcome and SNPs that are independent of each other. Simulation II aimed at estimating the success rate of our proposed QMDR with a quantitative outcome and two functional interacting SNPs embedded within a set of 18 independent SNPs.

#### Simulation I

The goal of simulation I was to study the testing score’s null distribution. To this end, we let the SNP number m = {10, 20, 50} and the sample size n = {200, 400, 800, 1600}. For each combination of m and n, we simulated m SNPs with a minor allele frequency drawn from a uniform distribution over the interval (0.1, 0.5) using Bernoulli distribution. We then simulated n continuous outcomes from a standard normal distribution. We simulated the SNP data and outcomes independently of each other so that there is no association between SNPs and outcome. We repeated this to create 2000 null datasets for each m by n combition. Thus, we created 24,000 total datasets. All simulations were carried out in R (http://cran.r-project.org/). We ran QMDR for each dataset and searched over all 1–4 way interactions. The testing score was obtained using a 10-fold cross-validation.

#### Simulation II

The goal of simulation II was to study the success rate of the proposed method. We also compared QMDR with the original MDR algorithm and GMDR to demonstrate the success rate gained by using the continuous outcome.

We first generated datasets based on different penetrance functions. We previously developed a total of 70 different penetrance functions that define a probabilistic relationship between the outcome and SNPs where the outcome is dependent on genotypes from two loci in the absence of any marginal effects [23]. These purely epistatic models were distributed evenly across seven broad-sense heritabilities (0.01, 0.02, 0.05, 0.1, 0.2, 0.3, and 0.4) and two different minor allele frequencies (0.2 and 0.4) using Bernoulli distribution, where all functional SNPs in the data set had either one or the other minor allele frequencies. A total of five models for each of the 14 heritability-allele frequency combinations were generated for a total of 70 models. We also vary the sample size to include sample size n = {400, 800, 1600}. Since the original outcome is case-control status and the penetrance function denotes the probability of being a case for each genotype combination, we make the following change to simulate continuous outcome using the same penetrance functions:

Let *f_ij_* be the element from the *i*th row and *j*th column of a penetrance function. and are the two functional interacting SNPs. The Quantitative outcome was simulated from a normal distribution
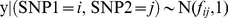
(2)


We repeated 100 times to get 100 datasets for each model.

### Real Data Analysis

The sample analyzed in this study was obtained from the ongoing prospective Prevention of Renal and Vascular End-Stage Disease (PREVEND) study [24]. The PREVEND study was designed to prospectively investigate the natural course of albuminuria and its relation to renal and cardiovascular disease in a large cohort drawn from the general population. Details of the study protocol have been described previously [25], [26]. In summary, during 1997–1998, all 85,421 inhabitants of the city of Groningen, the Netherlands, from the ages of 28 to 75 years old, were sent a one-page postal questionnaire. After exclusion of subjects with type 1 diabetes mellitus, females who were possibly pregnant, and males and females not able or willing to participate, a total of 6,000 subjects with a urinary albumin concentration≥10 mg l−1 and a random control sample of subjects with a urinary albumin concentration<10 mg l−1 (n = 2592) completed the screening protocol and formed the baseline PREVEND cohort (n = 8592). From this cohort, we selected a random sample of 2527 subjects (1338 females and 1189 males) as being representative of the entire population from which the PREVEND cohort was selected. These individuals were used in the present study to explore genetic predictors of tissue plasminogen activator (t-PA) and plasminogen activator inhibitor-1 (PAI-1) levels in the general population. We examined 7 polymorphisms from the renin-angiotensin, bradykinin and fribrinolytic systems that have been previously reported [24].

We carried out analysis in females and males separately since the distributions of t-PA and PAI-1 are gender specific. For each gender and expression combination, we first used an ANOVA and F-test to identify the main effect of each polymorphism. We filtered out the significant main effects via a two-step procedure: First, we fit a linear regression with PAI-1 or tPA plasma concentrations and all of the SNPs with a significant main effect (p<0.05). Secondly, we applied QMDR using the residual as the new quantitative trait to search over all possible two-way, three-way and four-way interactions. We used 10-fold cross-validation to determine the best overall model. We then ran a 10,000-fold permutation testing to estimate the *p-*value of the chosen model. Finally we compared the *p-*value obtained through permutation testing with that estimated using the empirical scores from simulation I.

## Results

### Simulation I Results

**Figure 1:**
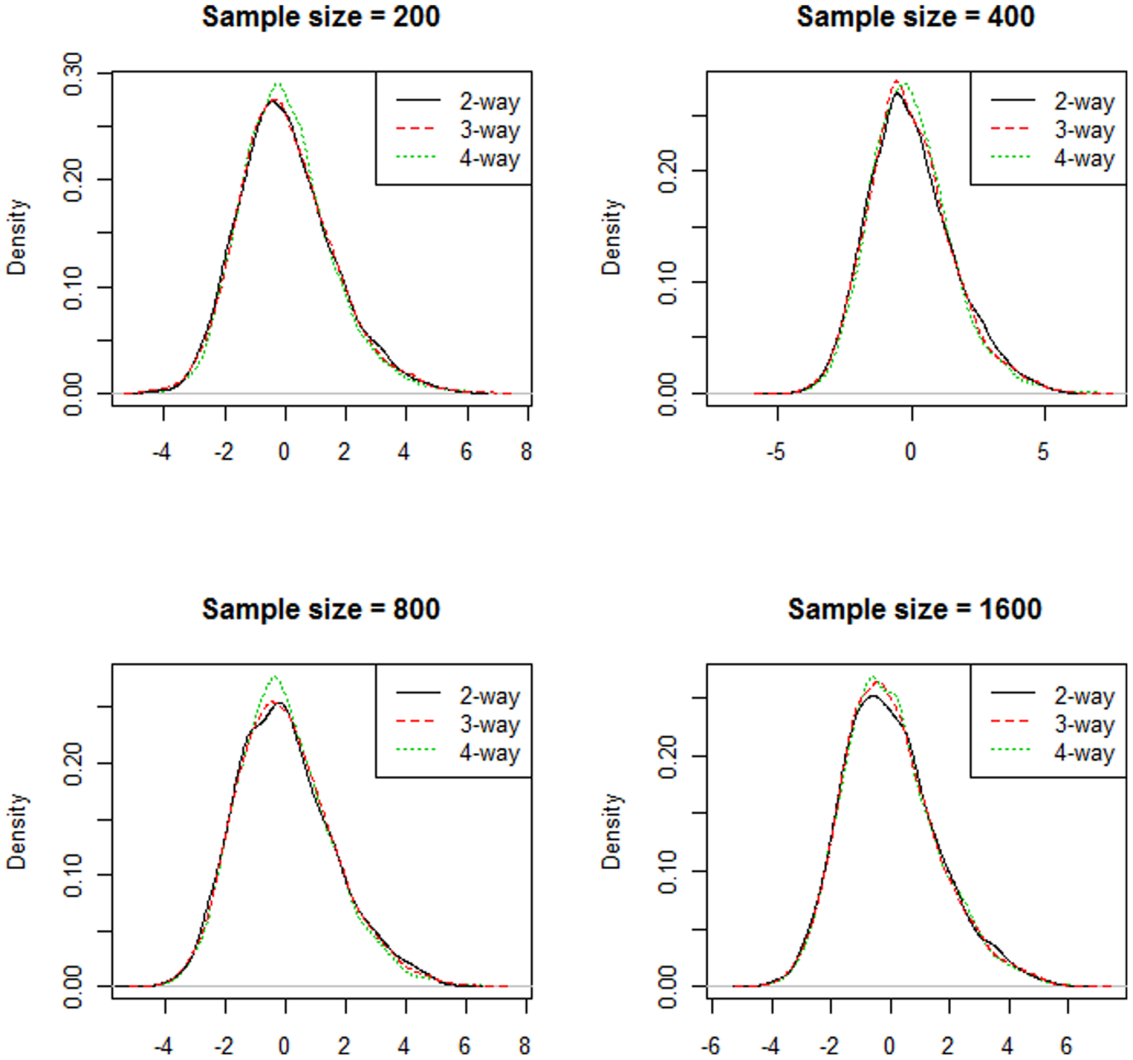
Empirical distribution of the 10-fold cross-validated testing score. The three curves on each figure represent the testing score from 2-way, 3-way and 4-way models.

The testing scores were expected to follow a standard normal distribution, since when the sample size is large (n >50), the T-statistics are asymptotically standard normal. However, from figure 1, the empirical distributions were not exactly standard normal. The estimated standard deviation was 1.6 with a slight right skew. We believe that this deviation was due to extra variation in the cross-validation procedure that resulted from the overlap among the training sets.

**Figure 2:**
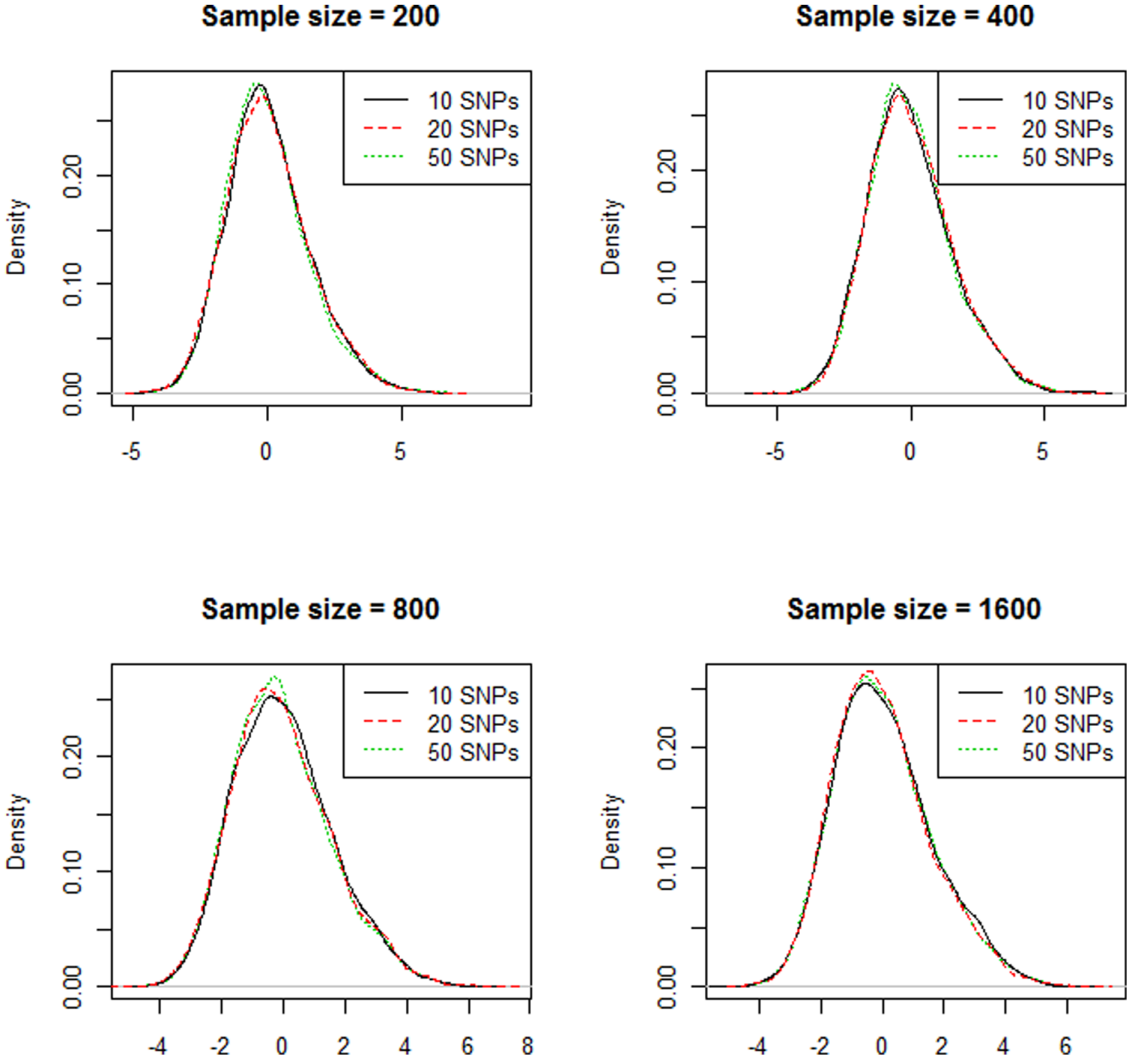
Empirical distribution of the 10-fold cross-validated testing score. The three curves on each figure represent testing scores from datasets with 10, 20, and 50 SNPs.

On the other hand, as seen in figure 1 and 2, we can tell that the null distributions are similar across different sample sizes, number of SNPs and interaction orders. The right tail regions, in particular, show almost perfect overlap. This indicates that we can use the 95^th^ quantile of the empirical distribution as a threshold to eliminate weak QMDR models, which is exactly what permutation testing can offer. Since the null distributions are “invariant”, we can potentially save a lot of computing time by comparing the testing score with a pre-calculated empirical distribution. In table 1, we used the 95th quantile of datasets with 400 samples to estimate the type I error. The estimated type I error was tightly distributed around 5% with a range from 4.1% to 6.2%.

**Table 1:** Estimated type I error using the 95th quantile of the testing scores from datasets with 400 samples.

*Type I error rate*	*n = 200*	*n = 400*	*n = 800*	*n = 1600*
2-way				
m = 10	4.8%	5.4%	5.8%	5.9%
m = 20	5.2%	5.1%	6.1%	5.5%
m = 50	5.5%	5.3%	5.8%	6.2%
3-way				
m = 10	4.8%	4.1%	5.6%	6.2%
m = 20	5.3%	5.8%	5.9%	5.3%
m = 50	4.2%	4.4%	6.1%	5.4%
4-way				
m = 10	4.1%	4.0%	4.9%	6.3%
m = 20	4.5%	4.8%	4.9%	4.5%
m = 50	4.1%	4.3%	4.6%	4.7%

### Simulation II Results

For each dataset, we used a median cut off to change the continuous outcome to a dichotomous outcome for analysis with the original MDR.

We applied MDR, GMDR and QMDR to search for the best one-way, two-way and three-way interaction model. The overall best model is determined by testing score and CV consistency. We counted the number of times that a SNP pair was correctly identified and divided that number by the total number of datasets (1000 for this simulation) to get the estimated success rate, as shown in table 2. QMDR and GMDR is generally more powerful than MDR. QMDR performs better than GMDR when the sample size is high or the heritability is above 0.1. However, GMDR performs better in low sample size and low heritability settings. One interesting thing to note is that GMDR’s success rate increase very slowly (ranging from 82% to 83%) at heritability 0.4 as sample size increases. The reason is that GMDR use balanced accuracy to rank interaction models. The variance of the quantitative outcome is ignored at this stage. The computing time for 100 datasets for GMDR is 7.9 min vs 4.4 min for QMDR. This indicates that QMDR is more efficient than GMDR.

**Table 2:** Success rate table for MDR, GMDR and QMDR.

*Success rate*	*h = 0.4*	*h = 0.3*	*h = 0.2*	*Heritability* *h = 0.1*	*h = 0.05*	*h = 0.02*	*h = 0.01*
**n = 400**							
QMDR	80.7%	69.3%	42.0%	12.7%	2.2%	0.6%	0.2%
GMDR	81.9%	73.2%	46.1%	15.2%	3.8%	0.8%	0.6%
MDR	66.4%	50.4%	26.7%	7.6%	1.8%	0.7%	0.6%
**n = 800**							
QMDR	85.4%	83.7%	74.6%	41.1%	8.6%	1.7%	0.7%
GMDR	83.1%	83.0%	76.0%	43.9%	10.0%	1.5%	0.8%
MDR	85.0%	79.8%	60.6%	26.1%	4.7%	1.1%	0.4%
**n = 1600**							
QMDR	83.4%	80.1%	78.0%	75.0%	25.6%	5.3%	1.3%
GMDR	82.2%	77.6%	75.7%	76.5%	30.5%	6.5%	1.8%
MDR	81.9%	80.1%	78.6%	58.1%	15.8%	3.7%	1.2%

### Real Data Results

The ANOVA for PAI-1 revealed a significant main effect from PAI 4G/5G with F-test *p-*value of 0.0006 for female and 0.002 for male. This verified previous findings [24], [27]. For tPA, we found a significant main effect of ATR1AC in females only (p = 0.04) and none in males.

After adjusting out the main effect of PAI 4G/5G and ATR1AC using residuals, we ran QMDR to identify interaction models. In table 3, we list the best interaction models identified from 10-fold cross-validation for PAI-1 and tPA expression in males and females. The best model for PAI-1 in females is a four-way interaction of BR2_58CT, ATR1AC, ACEID and BRB2EX1. The 10,000 permutation testing revealed a significant *p-*value of 0.014. Since we also found a significant main effect of PAI 4G/5G, we ran a linear regression model with PAI 4G/5G and an interaction between BR2_58CT, ATR1AC, ACEID and BRB2EX1 as the predictors and found that those 5 SNPs could explain 9.5% of the total variance of PAI-1 in females. Considering the sample size (>1000) and the continuous outcome, this indicated a strong association between PAI-1 and the 5 SNPs in females. The best interaction models from the other 3 datasets were not significant as determined by the permutation testing. One interesting thing to note was that removing the main effect did not exclude it from being chosen in the best interaction model. For example, PAI 4G/5G’s main effect was removed from PAI-1 in males and the best interaction model is four-way interactions of PAI 4G/5G, BR2_58CT, ACEID and BRB2EX1. We note that the amount of variance explained by the significant model was much better than previously reported using other analytical methods.

**Table 3:** Best overall model identified by QMDR.

File	Best model	CV testing score	Empirical P-value	Permutated P-value
**PAI (Female)**	BR2_58CT & ATR1AC & ACEID & BRB2EX1	**3.46**	**0.018**	**0.014**
**PAI (Male)**	PAI4G5G & BR2_58CT & ACEID & BRB2EX1	**0.98**	**0.244**	**0.259**
**tPA (Female)**	BR2_58CT & ACEID	**1.60**	**0.152**	**0.167**
**tPA (Male)**	PAI4G5G & BR2_58CT & BRB2EX1	**1.11**	**0.222**	**0.241**

The empirical *p-*values were very close (difference less than 2%) to those obtained from permutation tests. For *p-*values that are within the critical region, the empirical p-values are more accurate (1.4% vs 1.8%). This suggests that we can rely on the empirical distribution from simulations to estimate the significance of QMDR models, potentially saving months of computation time in genome-wide association studies.

## Discussion

In this paper, we presented a novel algorithm to identify SNP interactions associated with a quantitative outcome. There are two unique contributions from this paper: first of all, we offer a computationally efficient algorithm, QMDR, to identify epistatic models that are associated with a quantitative outcome. We have distributed free Java-based software for this QMDR on sourceforge.net. Secondly, we present a testing score that is “invariant” for all sample sizes, number of SNPs and interaction orders. Simulations and real data analyses both showed that the empirical *p-*value is very close to permutated *p-*value thereby permitting a significant reduction in computing time.

QMDR changes the representation of the data by pooling different genotype combinations into a two-level single attribute. This challenges the traditional analysis method of using many dummy variables to represent every genotype combination. Due to limitations in sample size, some of the dummy variables used in traditional models can be represented by very sparse or no data. This makes the modelling of high-order combinations very difficult. QMDR solves this dilemma elegantly by collapsing all high-level genotypes and all low-level genotypes into a two level attribute. This not only makes it easier to detect higher order interaction terms, but also makes it possible to incorporate the QMDR attribute into other statistical and machine learning algorithms, such as Boosting [28] and neural networks. This will allow researchers to build more accurate models that involve multiple genotype combinations. As we can see from real data results, QMDR successfully identifies a four-way interaction model that is strongly associated with female PAI-1 levels. This is good example of how QMDR can gain biological information above that previously obtained using a more traditional ANOVA approach [24].

QMDR also offers a cross-validation procedure to pick the best model based on the testing score. Since high-order interactions tend to have better training scores than low-order interactions, these cross-validation procedures are necessary to limit model over-fitting. The QMDR algorithm also explores the empirical distribution of the testing score from the null models and applies it to estimate the significance of the selected model. From the simulation, we demonstrated that QMDR can detect the presence of multiplicative interaction models, even when main effects are not statistically significant. These epistatic combinations tend to be missed or dropped when using traditional linear regression approaches. Last but not least, QMDR is computationally efficient. One of the first algorithms developed to explore gene-gene interactions was the Combinatorial Partitioning Method (CPM) [18]. While elegant, CPM’s functionality is limited by its inefficiency; its computing time increase exponentially with the number of genes examined 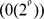. In contrast, QMDR’s computing time is 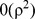 for two-way interactions. QMDR is has a nearly two-fold computational speed advantage over GMDR, as demonstrated through simulations. This increase in speed is vitally important and makes QMDR a feasible method to use when exploring large datasets such as genome-wide association studies.

Despite the advantages stated above, one limitation to the approach is that the QMDR method does not have a way to adjust for covariate effects such as age, gender and smoking status, an often necessary step to obtain an unconfounded SNP interactions-outcome association. Investigators can, however, first fit a model with potential confounders and the outcome of interest and then use the residuals in QMDR, as we did to adjust for SNP main effects in this paper. However, if there are too many covariates, this approach may overfit the data and sometimes fail due to limited sample size. One strength of QMDR is that it can be extended to the analysis of interactions among non-genetic variables as well as gene-environment interactions. For example, QMDR will facilitate pharmacogenomic analyses by identifying combinations of drug treatments and genotypes that affect time to progression.

In summary, we demonstrate that QMDR is a promising dimension reduction method for the efficient identification of SNP interactions. We believe that it will play an important role as part of a research strategy to understand genetic influences on disease outcomes that embraces the complexity of the genotype-phenotype mapping relationship.

We have uploaded Java-based QMDR software as part of MDR 3.0 to: http://sourceforge.net/projects/mdr/.
